# Trends in Health Information Technology Use Among the US Population With and Without Cardiovascular Risk Factors, 2012-2018: Evidence From the National Health Interview Survey

**DOI:** 10.2196/29990

**Published:** 2021-09-30

**Authors:** Nikhila Gandrakota, Mohammed K Ali, Megha K Shah

**Affiliations:** 1 Department of Family & Preventive Medicine Emory University School of Medicine Atlanta, GA United States; 2 Hubert Department of Global Health Rollins School of Public Health Emory University Atlanta, GA United States

**Keywords:** telemedicine, cardiovascular risk factors, health information technology, telehealth, digital health, public health, surveillance

## Abstract

**Background:**

The COVID-19 pandemic has required clinicians to pivot to offering services via telehealth; however, it is unclear which patients (users of care) are equipped to use digital health. This is especially pertinent for adults managing chronic diseases, such as obesity, hypertension, and diabetes, which require regular follow-up, medication management, and self-monitoring.

**Objective:**

The aim of this study is to measure the trends and assess factors affecting health information technology (HIT) use among members of the US population with and without cardiovascular risk factors.

**Methods:**

We used serial cross-sectional data from the National Health Interview Survey for the years 2012-2018 to assess trends in HIT use among adults, stratified by age and cardiovascular risk factor status. We developed multivariate logistic regression models adjusted for age, sex, race, insurance status, marital status, geographic region, and perceived health status to assess the likelihood of HIT use among patients with and without cardiovascular disease risk factors.

**Results:**

A total of 14,304 (44.6%) and 14,644 (58.7%) participants reported using HIT in 2012 and 2018, respectively. When comparing the rates of HIT use for the years 2012 and 2018, among participants without cardiovascular risk factors, the HIT use proportion increased from 51.1% to 65.8%; among those with one risk factor, it increased from 43.9% to 59%; and among those with more than one risk factor, it increased from 41.3% to 54.7%. Increasing trends in HIT use were highest among adults aged >65 years (annual percentage change [APC] 8.3%), who had more than one cardiovascular risk factor (APC 5%) and among those who did not graduate from high school (APC 8.8%). Likelihood of HIT use was significantly higher in individuals who were younger, female, and non-Hispanic White; had higher education and income; were married; and reported very good or excellent health status. In 2018, college graduates were 7.18 (95% CI 5.86-8.79), 6.25 (95% CI 5.02-7.78), or 7.80 (95% CI 5.87-10.36) times more likely to use HIT compared to adults without high school education among people with multiple cardiovascular risk factors, one cardiovascular risk factor, or no cardiovascular risk factors, respectively.

**Conclusions:**

Over 2012-2018, HIT use increased nationally, with greater use noted among younger and higher educated US adults. Targeted strategies are needed to engage wider age, racial, education, and socioeconomic groups by lowering barriers to HIT access and use.

## Introduction

The COVID-19 pandemic has significantly changed ambulatory care delivery, which has likely impacted the ability of adults living with cardiovascular disease (CVD) risk factors to manage their health conditions. Factors including shortages of testing supplies, personal protective equipment, state and health system mandates, and difficulty maintaining adequate staffing led to most providers deferring elective and annual physical examinations [1,2] or adapting to telemedicine to decrease the spread of the virus [2-4]. Patients have also avoided in-person visits due to the risk of exposure [4]. Further local and state recommendations, promoting social distancing, have also influenced adults seeking care for chronic diseases. Studies show that in-person outpatient visits dropped by almost 60% early in the pandemic [5]. Thus, regular follow-up with clinicians for care of chronic conditions has likely been delayed or forgone.

Most recent estimates suggest almost half of the US population report having one CVD risk factor, such as obesity, high blood pressure, or diabetes [6]. To prevent disease progression and reduce the risk of complications, these conditions require regular self-management (ie, numerous daily decisions on diet, exercise, and medication use) and follow up with their clinicians for continued health education and medication titration [7].

The current environment has provided an opportunity for a digital revolution in health care, with unparalleled, rapid expansion of telehealth and telemedicine. Previous literature showed that 74% of American adults access the internet, 57% of American households have broadband connections, and 61% of adults obtain health information on the web [8]. However, the extent to which Americans living with CVD risk factors access and use digital technology and their ability to do so are unknown. It also remains unclear which demographic groups and other subgroups of American adults with CVD risk factors access health technology. Using nationally representative data, we examined trends in health information technology (HIT) use in the years prior to the COVID-19 pandemic. We compared adults with and without CVD risk factors in the last decade, and we examined which Americans were at highest risk of limited digital access.

## Methods

### Data Source

We used data from the National Health Interview Survey (NHIS) from 2012 to 2018. The NHIS is an annual survey that collects health-related information on a representative sample of the noninstitutionalized population of the United States [9]. The National Center for Health Statistics oversees the annual cross-sectional collection of NHIS data. NHIS samples approximately 45,000 households and 110,000 persons every year. The survey uses a 3-stage stratified cluster-probability sampling design, and all data are self-reported. One adult from each sampled household is randomly selected to provide detailed information on health indicators, social characteristics, and demographics. The annual response rates for the NHIS were 77.6%, 75.7%, 73.8%, 70.1%, 67.9%, 66.5%, and 64.2% of the eligible households in the sample for the years 2012-2018, respectively. More details of the NHIS sampling procedures are reported elsewhere [10]. This study was considered to be exempt by the Emory University Institutional Review Board.

### Measures

We used HIT use questions from the years 2012-2018 for the study. Respondents were asked, “During the past 12 months, have you ever used computers for any of the following: (1) to look up health information on the Internet, (2) to fill a prescription, (3) to schedule a web-based appointment with a health care provider, (4) to communicate with a health care provider by email?” If an individual indicated use for any of these four purposes, they were considered to have used HIT in the past 12 months. Participants were classified as “Used HIT for a general purpose” if they looked up health information on the internet and as “Used HIT for a clinical purpose” if they filled a prescription on the web, scheduled a web-based appointment, or communicated with a health care provider by email.

The CVD risk factors included in the study were self-reported diabetes, hypertension, hyperlipidemia, and obesity, as these are the most common conditions at risk for heart disease [11]. We identified the participants as having one or more of the four CVD risk factors when they responded yes to the question “Have ever been told by a doctor or health care provider that you have hypertension/diabetes/high cholesterol?” or obesity, defined as a reported BMI classified as overweight (25.0-29.9 kg/m^2^) or obese (>30.0 kg/m^2^) [12]. We stratified the population into adults with no CVD risk factors, one CVD risk factor, and multiple CVD risk factors for the CVD risk factors mentioned above.

We examined a range of household-, individual-, and health-related factors expected to impact HIT use. Individual-level characteristics included race/ethnicity (Hispanic, non-Hispanic White, non-Hispanic, Black, non-Hispanic Asian, and non-Hispanic all other race groups), insurance type (uninsured, insured–private or public), age (18-25, 26-44, 45-64, and ≥65 years), education (<high school, high school graduate, some college, and college graduate) and sex (male, female). Household-level characteristics included marital status (married and unmarried), geographic region (Northeast, Midwest, South, and West) and poverty. Poverty was determined using the poverty income ratio variable in NHIS, which measures the ratio of the annual family income divided by the household-adjusted federal poverty level in dollars, as defined by the Census Bureau for that survey year [13]. This variable was recoded as in poverty/near poverty for ratios <2.00 and not in poverty/near poverty for ratios ≥2.00. Health-related factors included an indicator variable on perceived health status (poor, fair, good, very good, and excellent), as prior evidence suggests that a poor perceived health status might decrease the likelihood of HIT use [14]. English proficiency of the adults was classified into two categories: not at all/not well and well/very well. However, this information was only available for the year 2018.

### Statistical Analysis

The unit of analysis was the individual. Sampling weights (assigned by the NHIS) were used to account for uneven data collection probabilities stemming from the NHIS sample design and nonresponse. SAS, version 9.4 (SAS Institute) was used for the analyses. Sampling weights were used to obtain nationwide representative estimates and standard errors because NHIS uses a multistage probability complex sampling design that incorporates stratification, clustering, and oversampling of some subpopulations (eg, Black, Hispanic, and Asian). Weighted means along with 95% confidence intervals are reported for all continuous variables.

The proportion of HIT use among respondents by CVD risk status (no risk factors, one risk factor, or multiple CVD risk factors) were compared for the years 2012 and 2018 using chi-square tests. We also compared characteristics of the respondents with and without HIT use for the years 2012 and 2018. Among HIT users, the proportions using the internet for clinical use and general use were also compared for the years 2012 and 2018.

Using linear trend analysis, we then compared adults by CVD risk factor status, highest level of education, and age groups of 18-25, 26-44, 45-64, and ≥65 years to examine HIT use trends over the years 2012-2018. The annual percentage changes (APCs) of HIT use were calculated for each of the age, CVD risk factor, and education groups.

Independent predictors of HIT use were identified using multiple logistic regression models adjusted for age and sex for each of the risk factor groups of one CVD risk factor condition, multiple CVD risk factors, and no CVD risk factors for the years 2012 and 2018.

## Results

### Demographics

Among a total of 58,992 respondents in the years 2012 (n=33,885) and 2018 (n=25,107), males comprised 45.5% of the total respondents in both 2012 and 2018. In 2012, 69.4% of the total respondents were non-Hispanic White and 12.5% were non-Hispanic Black, while in 2018, 66.9% of the total respondents were non-Hispanic White and 12.7% were non-Hispanic Black (Table 1). In 2012, 26% of people had no CVD risk factors, 37% had one CVD risk factor, and 37% had more than one CVD risk factor. In 2018, just over 20% had no CVD risk factors, 37% had one CVD risk factor, and 40% had more than one CVD risk factor.

**Table 1:** General characteristics of the National Health Interview Survey populations in 2012 (n=33,885) and 2018 (n=25,107).

		Values, n (%)								
		2012					2018			
		Used HIT^a^	Used HIT for a general purpose^b^	Used HIT for a clinical purpose^c^	Did not use HIT	Used HIT		Used HIT for a general purpose	Used HIT for a clinical purpose	Did not use HIT
**Age (years)**										
	18-25	1856 (47.7)	1788 (45.9)	346 (8.9)	2039 (52.3)	1371 (63.1)		1314 (60.5)	513 (23.6)	801 (36.9)
	26-44	5597 (50.3)	5386 (48.4)	1403 (12.6)	5524 (49.7)	5050 (68.5)		4791 (65.0)	2303 (31.2)	2323 (31.5)
	45-64	5087 (43.8)	4825 (41.5)	1506 (13.0)	6534 (56.2)	5126 (61.5)		4764 (57.1)	2505 (30.0)	3209 (38.5)
	≥65	1764 (24.3)	1622 (22.4)	555 (7.7)	5484 (75.7)	3097 (42.9)		2808 (38.9)	1443 (20.0)	4130 (57.1)
**Sex**										
	Male	5522 (36.8)	5218 (34.8)	1488 (9.9)	9476 (63.2)	6121 (53.6)		5632 (49.3)	2723 (23.8)	5299 (46.4)
	Female	8782 (46.5)	8403 (44.5)	2322 (12.3)	10,105 (53.5)	8523 (62.3)		8045 (58.8)	4041 (29.5)	5164 (37.7)
**Ethnicity**										
	Hispanic	1601 (27.7)	1524 (26.4)	353 (6.1)	4172 (72.3)	1407 (44.7)		1330 (42.3)	514 (16.3)	1740 (55.3)
	Non-Hispanic White	9956 (48.6)	9498 (46.4)	2734 (13.4)	10,518 (51.4)	10,905 (62.8)		10,181 (58.6)	5237 (30.1)	6472 (37.2)
	Non-Hispanic Black	1654 (32.1)	1564 (30.3)	391 (7.6)	3502 (67.9)	1378 (47.2)		1282 (43.9)	577 (19.7)	1543 (52.8)
	Non-Hispanic Asian	969 (45.8)	915 (43.2)	308 (14.6)	1147 (54.2)	789 (60.7)		727 (56.0)	392 (30.1)	510 (39.3)
	Non-Hispanic all other race groups	124 (33.9)	120 (32.8)	24 (6.5)	242 (66.1)	165 (45.5)		157 (43.3)	44 (12.1)	198 (54.5)
**Educational status**										
	<High school	958 (14.9)	909 (14.1)	149 (2.3)	5483 (85.1)	927 (26.7)		860 (24.8)	266 (7.7)	2541 (73.3)
	High school graduate	2232 (29.0)	2109 (27.4)	462 (6.0)	5466 (71.0)	2242 (41.5)		2073 (38.4)	797 (14.7)	3163 (58.5)
	Some college	5004 (48.2)	4774 (45.9)	1209 (11.6)	5387 (51.8)	4730 (62.5)		4412 (58.3)	2012 (26.6)	2835 (37.5)
	College graduate	5792 (65.8)	5528 (62.8)	1861 (21.2)	3010 (34.2)	6366 (78.2)		5972 (73.4)	3466 (42.6)	1770 (21.8)
**Poverty**										
	In poverty/near poverty	3415 (30.3)	3302 (29.3)	641 (5.7)	7852 (69.7)	2774 (44.5)		2625 (42.1)	920 (14.7)	3466 (55.5)
	Not in poverty/near poverty	9047 (53.4)	8555 (50.5)	2756 (16.3)	7883 (46.6)	10,023 (66.5)		9334 (61.9)	5061 (33.6)	5045 (33.5)
**Marital status**										
	Unmarried	6486 (38.1)	6188 (36.3)	1625 (9.5)	10,548 (61.9)	6470 (52.9)		6050 (49.4)	2750 (22.4)	5769 (47.1)
	Married	7794 (46.5)	7410 (44.2)	2181 (13.0)	8982 (53.5)	8152 (63.6)		7606 (59.3)	4004 (31.2)	4669 (36.4)
**Region**										
	Northeast	2486 (43.9)	2389 (42.2)	575 (10.1)	3181 (56.1)	2419 (59.3)		2286 (56.0)	1050 (25.7)	1661 (40.7)
	Midwest	3119 (44.3)	2982 (42.4)	792 (11.2)	3921 (55.7)	3481 (59.1)		3262 (55.4)	1593 (27.0)	2411 (40.9)
	South	4691 (38.1)	4463 (36.3)	1171 (9.5)	7614 (61.9)	5074 (55.2)		4740 (51.6)	2342 (25.5)	4110 (44.8)
	West	4008 (45.2)	3787 (42.7)	1272 (14.3)	4865 (54.8)	3670 (61.7)		3389 (56.9)	1779 (29.9)	2281 (38.3)
**Insurance**										
	Uninsured	1947 (32.3)	1917 (31.8)	223 (3.7)	4082 (67.7)	987 (45.4)		960 (44.1)	234 (10.7)	1188 (54.6)
	Public	2387 (28.8)	2280 (27.5)	567 (6.8)	5913 (71.2)	3355 (45.8)		3096 (42.2)	1392 (19.0)	3975 (54.2)
	Private	9935 (51.1)	9390 (48.3)	3014 (15.5)	9511 (48.9)	10,265 (66.1)		9585 (61.8)	5130 (33.0)	5255 (33.9)
**Perceived health status**										
	Excellent	4104 (47.1)	3916 (44.9)	1050 (12.0)	4612 (52.9)	3988 (63.8)		3732 (59.7)	1772 (28.3)	2265 (36.2)
	Very good	5185 (49.1)	4939 (46.7)	1416 (13.4)	5380 (50.9)	5311 (63.8)		4977 (59.8)	2541 (30.5)	3010 (36.2)
	Good	3586 (37.9)	3386 (35.8)	960 (10.2)	5867 (62.1)	3748 (54.0)		3474 (50.0)	1719 (24.8)	3192 (46.0)
	Fair	1151 (29.4)	1118 (28.6)	299 (7.7)	2759 (70.6)	1263 (45.9)		1184 (43.1)	584 (21.2)	1488 (54.1)
	Poor	273 (22.2)	258 (21.0)	83 (6.8)	954 (77.8)	330 (39.6)		306 (36.7)	147 (17.6)	503 (60.4)
**English proficiency**										
	Not good/none	No information	No information	No information	No information	183 (17.0)		170 (15.8)	38 (3.5)	893 (83.0)
	Very good/ good	No information	No information	No information	No information	14,460 (60.2)		13,506 (56.2)	6725 (28.0)	9567 (39.8)
**Cardiometabolic risk status**										
	No risk factors	3946 (49.1)	3817 (47.5)	936 (11.6)	4096 (50.9)	3414 (65.5)		3269 (62.7)	1512 (29.0)	1798 (34.5)
	One risk factor	4984 (41.6)	4761 (39.7)	1195 (10.0)	7009 (58.4)	5005 (59.2)		4709 (55.7)	2145 (25.3)	3453 (40.8)
	Multiple risk factors	4597 (38.9)	4308 (36.5)	1493 (12.6)	7214 (61.1)	5227 (54.1)		4761 (49.3)	2611 (27.0)	4438 (45.9)

^a^HIT: health information technology.

^b^Used HIT for general purposes: looked up health information on the internet.

^c^Used HIT for clinical purposes: filled a prescription on the web, scheduled a web-based appointment with a health care provider, or communicated with a health care provider by email. Note: ^b^ and ^c^ are not mutually exclusive.

### Prevalence of HIT Use and CVD Risk Factors

In 2012, 41.6% of the total weighted sample of respondents looked up health information on the internet, representing 42.3 million Americans. Of those who used HIT, 6.8% filled a prescription on the internet, less than 5% made web-based appointments with their health care provider, and 5.8% communicated with their health care provider via email. In 2018, 54.2% of the total weighted sample of respondents looked up health information on the internet, representing 60.5 million. Approximately 11% filled a prescription on the internet, approximately 16% made web-based appointments with their health care provider, and 16.5% communicated with their health care provider via email (Table 1).

Overall, in 2012, 44.5% of the total weighted sample of respondents reported using HIT for any one of the four purposes listed above, representing 44.5 million, and in 2018, this proportion increased to 58.6%, representing 64.7 million.

Prevalence of HIT use among respondents without any CVD risk factors (weighted percentage 51.1%, 95% CI 49.8%-52.5%) was significantly higher than respondents with one CVD risk factor (weighted percentage 43.9%, 95% CI 42.8%-45%) or multiple CVD risk factors (weighted percentage 41.3%, 95% CI 40.1%-42.4%) in 2012. Although there was an increase in the prevalence of HIT use in 2018 among all the CVD risk groups compared to 2012, the highest use of HIT was still among respondents without any CVD risk factors (weighted percentage 65.8%, 95% CI 64.1%-67.4%) compared to respondents with one CVD risk factor (weighted percentage 60%, 95% CI 57.5%-60.4%) or multiple CVD risk factors (weighted percentage 54.7%, 95% CI 53.4%-55.9%). A detailed comparison of types of HIT use by respondents with and without CVD risk factors in the years 2012 and 2018 is shown in Table 2.

**Table 2:** Use of HIT by year for the general National Health Information Survey and CVD risk factor strata for 2012 and 2018.

HIT^a^ use	Value (%)^b^								
	2012 (n=33,885; N=99,819,805)					2018 (n=25,107; N=110,273,504)			
	All	No CVD^c^ risk factors	One CVD risk factor	Multiple CVD risk factors	All		No CVD risk factors	One CVD risk factor	Multiple CVD risk factors
Any health information technology use	44.5	51.1	43.9	41.2	58.6		65.8	60.0	54.7
Looked up health information on the internet	41.5	48.7	41.6	38.1	54.2		62.7	55.2	49.5
Filled a prescription on the internet	6.7	5.6	5.4	9.1	11.3		9.5	9.3	14.0
Scheduled a medical appointment on the internet	4.6	5.8	4.5	4.2	16.3		19.7	16.7	14.2
Communicated with a health care provider by email	5.8	6.4	5.7	6.0	16.4		17.7	15.0	17.2

^a^HIT: health information technology.

^b^All percentages are weighted.

^c^CVD: cardiovascular disease.

### Trend Analyses

**Figure 1:**
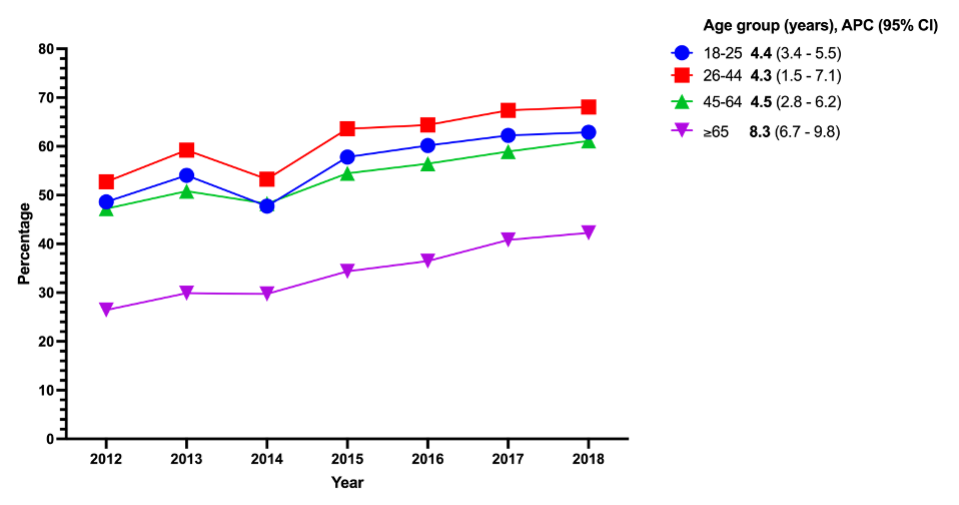
Trends in health information technology use by age, 2012-2018. APC: annual percentage change.

In the linear trend analysis stratified by CVD risk status, age, and education from 2012 to 2018 (Figure 1), the APC in HIT use from 2012 to 2018 increased by 4.4% (95% CI 3.4%-5.5%) in adults aged 18-25 years, 4.3% (95% CI 1.5%-7.1%) in the 26-44 years age group, 4.5% (95% CI 2.8%-6.2%) in the 45-64 years age group, and 8.3% (95% CI 6.7-9.8) in the ≥65 years age group.

**Figure 2:**
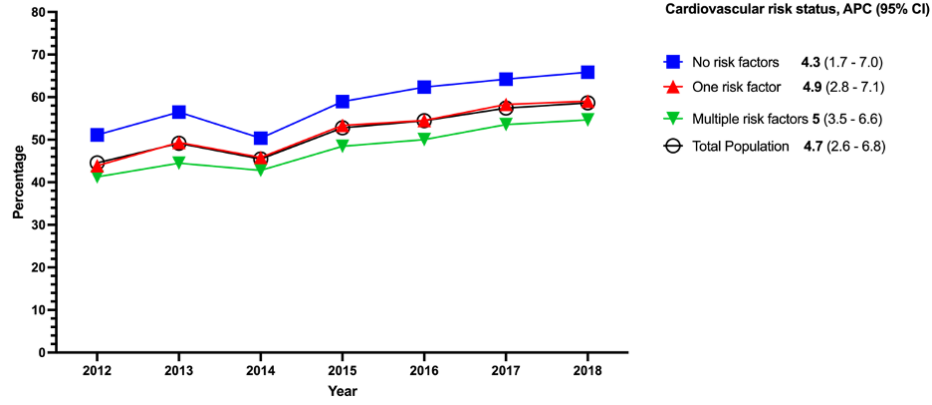
Trends in health information technology use by cardiovascular disease risk status, 2012-2018. APC: annual percentage change.

**Figure 3:**
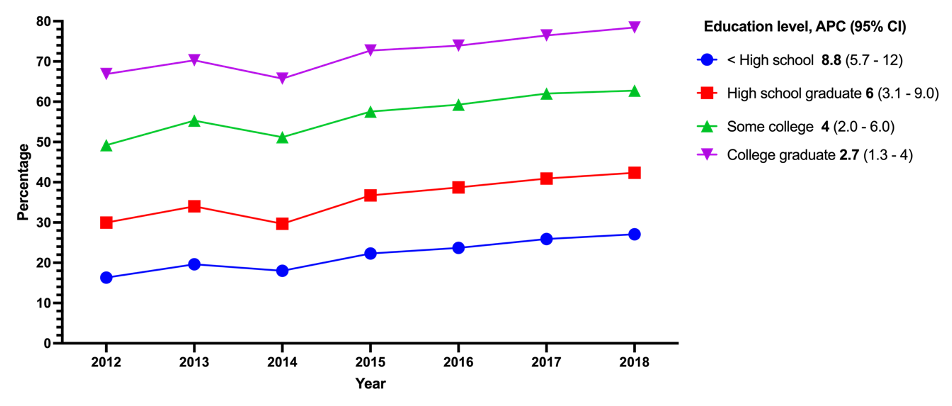
Trends in health information technology use by highest level of education, 2012-2018. APC: annual percentage change.

Respondents with none of the CVD risk factors were the highest HIT users (Figure 2); however, they had a smaller APC of 4.3% (95% CI 1.7%-7.0%) from 2012 to 2018. People with one CVD risk factor had an APC of 4.9% (95% CI 2.8%-7.1%), and those who had multiple CVD risk factors showed an APC of 5% (95% CI 3.5%-6.6%) from 2012 to 2018. The highest APC of 8.8% (95% CI 5.7%-12%) by education status was seen among people who had not graduated from high school (Figure 3).

### Characteristics Associated With HIT Use Among Adults With and Without CVD Risk Factors

In 2012, among those with multiple CVD risk factors, college graduation was associated with the highest odds of HIT use (odds ratio [OR] 8.59, 95% CI 7.03-10.50) compared to adults without high school education. This gap remained in 2018, when college graduates were over 7 times more likely to use HIT (OR 7.18, 95% CI 5.86-8.79) than adults without high school education. Similar associations were seen in those with a single CVD risk factor or no CVD risk factors (Table 3).

**Table 3:** Factors associated with health information technology use by CVD risk status: results from multivariable logistic regression for 2012 and 2018.

			Odds ratio (95% CI)								
		2012						2018			
		Multiple CVD risk factors (n= 35,133,127)		One CVD risk factor (n= 35,057,617)	No CVD risk factors (n= 24,644,831)	All (n= 94,835,575)	Multiple CVD risk factors (n= 40,877,272)		One CVD risk factor (n= 38,466,418)	No CVD risk factors (n= 23,891,951)	All (n= 103,235,641)
**Age (years; reference: 18-25)**											
	26-44	0.88 (0.59-1.32)		0.91 (0.76-1.08)	*0.82 (0.68-0.98)* ^a^	*0.86 (0.77-0.96)*	0.84 (0.52-1.36)		0.89 (0.72-1.09)	0.93 (0.75-1.15)	0.92 (0.80-1.05)
	45-64	*0.53 (0.35-0.78)*		*0.64 (0.54-0.77)*	*0.59 (0.49-0.71)*	*0.63 (0.57-0.71)*	*0.48 (0.30-0.76)*		*0.67 (0.54-0.83)*	*0.59 (0.48-0.74)*	*0.65 (0.57-0.74)*
	≥65	*0.22 (0.15-0.33)*		*0.28 (0.22-0.35)*	*0.21 (0.16-0.29)*	*0.27 (0.24-0.31)*	*0.20 (0.12-0.33)*		*0.29 (0.23-0.37)*	*0.25 (0.19-0.32)*	*0.29 (0.25-0.33)*
**Sex (reference: male)**											
	Female	*1.79 (1.60-2.00)*		*2.12 (1.91-2.36)*	*1.79 (1.56-2.05)*	*1.86 (1.75-1.98)*	*1.53 (1.38-1.70)*		*2.13 (1.89-2.41)*	*1.67 (1.41-1.98)*	*1.75 (1.63-1.88)*
**Race/ethnicity (reference: non-Hispanic White)**											
	Hispanic	*0.56 (0.47-0.67)*		*0.59 (0.50-0.69)*	*0.63 (0.53-0.75)*	*0.58 (0.52-0.63)*	*0.66 (0.54-0.80)*		*0.53 (0.45-0.62)*	*0.65 (0.52-0.82)*	*0.58 (0.52-0.64)*
	Non-Hispanic Black	*0.59 (0.51-0.70)*		*0.61 (0.52-0.71)*	*0.61 (0.50-0.75)*	*0.60 (0.55-0.66)*	*0.71 (0.60-0.83)*		*0.63 (0.53-0.76)*	*0.47 (0.36-0.63)*	*0.63 (0.56-0.71)*
	Non-Hispanic Asian	*0.54 (0.39-0.75)*		*0.82 (0.65-1.05)*	*0.70 (0.56-0.87)*	*0.68 (0.60-0.78)*	*0.60 (0.45-0.78)*		*0.60 (0.46-0.79)*	*0.52 (0.39-0.70)*	*0.56 (0.47-0.66)*
	Non-Hispanic all other race groups	0.70 (0.40-1.22)		0.92 (0.55-1.54)	0.97 (0.53-1.78)	0.81 (0.57-1.14)	0.79 (0.48-1.28)		0.59 (0.32-1.09)	0.45 (0.19-1.03)	*0.65 (0.46-0.93)*
**Educational status (reference: <high school)**											
	High school graduate	*1.73 (1.44-2.08)*		*1.90 (1.57-2.30)*	*1.50 (1.18-1.90)*	*1.74 (1.56-1.93)*	*1.55 (1.27-1.88)*		*1.44 (1.16-1.79)*	*1.83 (1.38-2.44)*	*1.59 (1.39-1.80)*
	Some college	*4.02 (3.34-4.84)*		*3.26 (2.74-3.89)*	*3.17 (2.54-3.95)*	*3.54 (3.21-3.91)*	*3.80 (3.18-4.55)*		*3.07 (2.51-3.76)*	*3.01 (2.23-4.06)*	*3.37 (2.98-3.82)*
	College graduate	*8.59 (7.03-10.50)*		*6.14 (5.07-7.43)*	*7.17 (5.63-9.10)*	*7.02 (6.30-7.82)*	*7.18 (5.86-8.79)*		*6.25 (5.02-7.78)*	*7.80 (5.87-10.36)*	*6.91 (6.07-7.86)*
**Poverty (reference: not in poverty)**											
	In poverty/near poverty	*0.57 (0.50-0.66)*		*0.65 (0.56-0.74)*	*0.84 (0.72-0.98)*	*0.67 (0.62-0.72)*	*0.61 (0.53-0.70)*		*0.83 (0.72-0.97)*	*0.83 (0.68-1.01)*	*0.73 (0.67-0.79)*
**Marital status (reference: unmarried)**											
	Married	*1.23 (1.10-1.38)*		1.10 (0.99-1.22)	1.08 (0.95-1.23)	*1.17 (1.10-1.25)*	*1.27 (1.13-1.42)*		*1.17 (1.04-1.31)*	*1.20 (1.01-1.43)*	*1.23 (1.15-1.31)*
**Region (reference: Northeast)**											
	Midwest	1.07 (0.88-1.30)		1.06 (0.88-1.28)	1.14 (0.95-1.38)	1.07 (0.96-1.20)	1.14 (0.96-1.35)		0.89 (0.73-1.10)	1.13 (0.85-1.50)	1.02 (0.90-1.16)
	South	1.01 (0.84-1.21)		0.97 (0.81-1.15)	0.89 (0.75-1.05)	0.95 (0.86-1.06)	1.12 (0.96-1.32)		0.93 (0.77-1.13)	0.87 (0.67-1.14)	1.00 (0.89-1.12)
	West	*1.01 (1.19-1.81)*		*1.31 (1.08-1.58)*	*1.32 (1.08-1.60)*	*1.34 (1.19-1.51)*	*1.39 (1.15-1.67)*		1.18 (0.96-1.46)	*1.32 (1.00-1.73)*	*1.27 (1.11-1.45)*
**Insurance (reference: uninsured)**											
	Public	1.03 (0.85-1.25)		0.89 (0.75-1.06)	1.17 (0.94-1.47)	1.04 (0.94-1.15)	1.27 (0.97-1.65)		1.11 (0.90-1.37)	1.20 (0.91-1.57)	*1.20 (1.05-1.37)*
	Private	*1.22 (1.00-1.48)*		*1.16 (1.00-1.35)*	*1.36 (1.13-1.62)*	*1.25 (1.13-1.38)*	*1.42 (1.08-1.87)*		*1.25 (1.02-1.52)*	1.21 (0.94-1.56)	*1.30 (1.14-1.49)*
**Perceived health status (reference: excellent)**											
	Very good	1.06 (0.89-1.27)		*1.34 (1.18-1.53)*	*1.26 (1.08-1.47)*	*1.32 (1.21-1.43)*	1.15 (0.96-1.39)		*1.38 (1.19-1.61)*	1.08 (0.91-1.27)	*1.26 (1.14-1.39)*
	Good	1.07 (0.91-1.25)		*1.19 (1.04-1.37)*	1.15 (0.97-1.37)	*1.24 (1.14-1.36)*	1.02 (0.84-1.24)		*1.24 (1.05-1.46)*	1.09 (0.88-1.34)	*1.21 (1.09-1.34)*
	Fair	1.05 (0.86-1.27)		*1.49 (1.17-1.89)*	1.00 (0.70-1.43)	*1.30 (1.15-1.47)*	1.17 (0.95-1.44)		*1.42 (1.12-1.79)*	0.93 (0.64-1.36)	*1.31 (1.16-1.49)*
	Poor	0.87 (0.64-1.17)		1.15 (0.74-1.77)	1.08 (0.53-2.21)	1.06 (0.86-1.29)	0.98 (0.74-1.29)		1.34 (0.85-2.12)	1.10 (0.57-2.11)	1.18 (0.97-1.43)

^a^Italic text indicates statistically significant results.

Among those respondents who reported no or multiple CVD risk factors, there was no difference in the odds of HIT use by health status. However, for those with one CVD risk factor, health status was associated with HIT use; in 2012, adults who reported their health status as “fair” were 1.49 times more likely to use HIT than adults who reported their health status as “excellent” (95% CI 1.17-1.89), and in 2018, they were 1.42 times more likely to do so (95% CI 1.12, 1.79). Overall, the significant predictors of HIT use were similar across all the three risk factor groups. In particular, after adjusting for health and sociodemographic factors, respondents who were relatively young, non-Hispanic White, female, and more educated; had private insurance and high income; and resided in the West were significantly more likely to be HIT users (Table 3).

## Discussion

### Principal Findings

HIT use increased by 10 to 15 percentage points in American adults over 2012-2018. Overall, the proportion of respondents using HIT for general purposes was greater than the proportion of people using HIT for clinical purposes in both 2012 and 2018. HIT users were more likely to be younger, female, and non-Hispanic White; have higher education and income; be married; and report their health status as very good or excellent.

The widespread, easy access to the internet for various purposes in recent times may have boosted the overall increasing trends of HIT use from 2012 to 2018 among all the risk factor groups [15]. Our findings show that in 2018, HIT use was the highest (66%) among adults with no CVD risk factors, followed by adults with one risk factor (59%); meanwhile, HIT use was the lowest among adults with multiple risk factors (55%). The lower use of HIT among respondents with multiple risk factors could be attributed to older age and disability. However, we found that the highest annual percentage change was seen among those with multiple CVD risk factors and those aged ≥65 years, which represents a positive change to address the potential digital divide by CVD risk status and older age, a known risk factor for limited digital access. The highest use of HIT among adults with no CVD risk factors may have been expected, because these groups also are likely to be younger [16]. A recent study [17] also demonstrated the rapid shift to telehealth during the COVID-19 pandemic; however, our findings demonstrate that the older sections of the population with multiple comorbidities may have been ill-equipped for this transition.

Our results also revealed wide variation in the odds of HIT use by individual, household-related, and health-related characteristics. Similar to previous studies, women had the highest odds of HIT use compared to men [18,19]. For example, in 2011, the US Centers for Disease Control and Prevention stated that women and adults aged 18-64 years belonging to higher income groups had the highest usage of the internet for health information than men and other age groups, respectively [20]. Socioeconomic factors, especially education, had higher influence on HIT use than health-related characteristics. Thus, despite widespread internet access in the United States, socioeconomic status disparities persist, suggesting the need for target strategies to improve HIT use/access.

Despite a significant recent increase in HIT use in the older population in recent times, a digital divide between younger and older persons persists [18,19,21]. Although there has been an increasing trend of HIT use among older adults, our findings reveal they have both the lowest use of HIT and also the highest rates of CVD risk factors [16]. Data from the Pew Research Center indicate that nationally, approximately 66% of adults aged over 65 years used the internet in the United States in 2018 [22]; however, our findings show that a much lower percentage of adults in this age group used HIT. Recent studies have shown that older adults are expressing a demand for HIT use [23] and would benefit the most from HIT use due to their comorbid conditions. Thus, studies and interventions are needed to increase HIT use for older adults, especially for clinical purposes. This could be achieved through designing easier technologies [24] to help older adults and those with hearing or visual impairments navigate HIT, as well as through clearing misconceptions and emphasizing the potential benefits of HIT use to improve care access [25].

The variations in HIT use related to race/ethnicity also deserve further attention. People who are White were more likely to use HIT than those in other race groups. A myriad of social and economic factors have likely created this divide, including the higher income, education, lifespan, and hence overall higher affordability and accessibility of HIT for White people compared to those in other race groups [26]. Chronic CVD-related disabilities, which are more common among other race groups than among White people [27,28], may create further barriers to digital access that could explain the lower proportions of HIT use among these groups. Language, cultural barriers, and access to care also influence the likelihood of HIT use among people of these races compared to White people [29,30], leading to the disparities we see in these findings. The perpetual racial and socioeconomic disparities in the digital divide [17] are a major public health concern as we continue to recover from the COVID-19 pandemic.

Among the types of HIT use, we observed that a majority reported seeking web-based health information compared to other types of use. This observation is in line with other studies showing that patients are increasingly relying on the internet as their primary source of answers to health-related questions [15,31,32]. Given the speed at which misinformation can spread on the internet, to ensure the credibility of health information obtained by patients, health systems and clinicians can play a key role in directing patients and HIT users to credible sources of information. This could include regular assessments at clinic visits of the sites where patients seek information on the web and provision of feedback or evidence-based resources for patients. Given the urgent need to use multiple methods to reach and improve access for patients during the ongoing pandemic, further investigation and interventions to address factors associated with low rates of HIT use for clinical purposes (to make appointments, email health care providers, fill prescriptions via internet) are needed.

### Strengths

Our study has several strengths. This study is the first to use nationally representative data to examine the prevalence of and factors associated with HIT use among people with and without CVD risk factors. The survey response rate is very good at 65-77%. Further, the study has a large sample size and was able to measure the trends through multiple years, from 2012-2018. To our knowledge, this study provides the first national assessment of HIT use among adults with CVD risk factors prior to the COVID-19 pandemic.

### Limitations

The general limitations of the NHIS data apply to this study as well. Firstly, the data are self-reported, and the questions pertaining to HIT use inquire about whether participants used HIT in the past 12 months, which means the responses could be subjected to recall bias. Second, the cross-sectional nature of the data limits the possibility to establish causal pathways between factors noted in our analysis and HIT use; cross-sectional data may also increase the risk of reverse causality. Further, the data lack information on English proficiency for the years 2012-2017. This is a limitation of the analysis, as English proficiency may have affected the rate of HIT use. Last, we are unable to quantify the amount of HIT use among the respondents, as some could be daily users and some could be monthly users; this may bear weight in the health access and knowledge of HIT tools.

### Conclusions

Our study provides a pre–COVID-19 assessment of HIT use among Americans with and without CVD risk factors. We found an increasing trend of HIT use among adults with and without CVD risk factors in the United States from 2012-2018. However, wide variation exists in use among Americans with CVD risk factors, who should be regularly accessing care. This variation has likely been exacerbated during the ongoing COVID-19 pandemic. Namely, older adults, racial and ethnic minority populations, and adults with multiple CVD risk factors are at high risk of having less access to HIT. A multipronged approach that includes education initiatives, affordable access to technology, and emphasis of health systems on creating platforms that all Americans can access are needed. Future studies to address these gaps are also needed to understand best practices.

## Abbreviations

APC: annual percentage change
CVD: cardiovascular disease
HIT: health information technology
NHIS: National Health Interview Survey
